# The Dynamics of Nucleotide Variants in the Progression from Low–Intermediate Myeloma Precursor Conditions to Multiple Myeloma: Studying Serial Samples with a Targeted Sequencing Approach

**DOI:** 10.3390/cancers14041035

**Published:** 2022-02-18

**Authors:** Bénedith Oben, Charlotte Cosemans, Ellen Geerdens, Loes Linsen, Kimberly Vanhees, Brigitte Maes, Koen Theunissen, Bert Cruys, Marta Lionetti, Ingrid Arijs, Niccolò Bolli, Guy Froyen, Jean-Luc Rummens

**Affiliations:** 1 Laboratory Experimental Hematology, Department Clinical Biology, Jessa Hospital, 3500 Hasselt, Belgium; charlotte.cosemans@uhasselt.be (C.C.); loes.linsen@uzleuven.be (L.L.); jean-luc.rummens@jessazh.be (J.-L.R.); 2 Faculty of Medicine and Life Sciences, Hasselt University, 3500 Hasselt, Belgium; kimberly.vanhees@jessazh.be (K.V.); ingrid.arijs@kuleuven.vib.be (I.A.); guy.froyen@jessazh.be (G.F.); 3 Centre for Environmental Sciences, Hasselt University, 3590 Diepenbeek, Belgium; 4 Laboratory Molecular Diagnostics, Department Clinical Biology, Jessa Hospital, 3500 Hasselt, Belgium; ellen.geerdens@jessazh.be (E.G.); brigitte.maes@jessazh.be (B.M.); bert.cruys@jessazh.be (B.C.); 5 Activity Center Biobanking, University Hospitals Leuven, 3000 Leuven, Belgium; 6 University Biobank Limburg (UBiLim), Clinical Biobank, Jessa Hospital, 3500 Hasselt, Belgium; 7 Department Hematology, Jessa Hospital, 3500 Hasselt, Belgium; koen.theunissen@jessazh.be; 8 Department Oncology and Hemato-Oncology, University of Milan, 20122 Milan, Italy; marta.lionetti@unimi.it (M.L.); niccolo.bolli@unimi.it (N.B.); 9 Laboratory for Translational Genetics, Department Human Genetics, University of Leuven, 3000 Leuven, Belgium; 10 Belgian Inflammatory Bowel Disease Research and Development (BIRD), 1930 Zaventem, Belgium; 11 Unità Operativa Complessa di Ematologia, Fondazione IRCCS Ca’ Granda Ospedale Maggiore Policlinico, 20122 Milan, Italy

**Keywords:** multiple myeloma, monoclonal gammopathy of undetermined significance, smoldering multiple myeloma, progression, targeted sequencing

## Abstract

**Simple Summary:**

Multiple myeloma (MM), characterized by the expansion of plasma cells in the bone marrow, is the second most common hematological malignancy. This incurable cancer is consistently preceded by non-malignant asymptomatic precursor conditions known as monoclonal gammopathy of undetermined significance (MGUS) and/or smoldering multiple myeloma (SMM). These pre-stages are relatively frequent, but only a select percentage of them will progress to MM. However, it is still not possible to individually predict when and which patients will develop MM. Therefore, this study aimed to investigate the mutational profile in the progression in serial bone marrow samples with a custom targeted sequencing panel, designed to detect variants in myeloma-related genes. Remarkably, almost all variants identified in the MM samples were also already present in the pre-stages, sometimes even many years before the progression. These results provide new important insights into the molecular mechanisms of the precursor conditions and progression to MM.

**Abstract:**

Multiple myeloma (MM), or Kahler’s disease, is an incurable plasma cell (PC) cancer in the bone marrow (BM). This malignancy is preceded by one or more asymptomatic precursor conditions, monoclonal gammopathy of undetermined significance (MGUS) and/or smoldering multiple myeloma (SMM). The molecular mechanisms and exact cause of this progression are still not completely understood. In this study, the mutational profile underlying the progression from low–intermediate risk myeloma precursor conditions to MM was studied in serial BM smears. A custom capture-based sequencing platform was developed, including 81 myeloma-related genes. The clonal evolution of single nucleotide variants and short insertions and deletions was studied in serial BM smears from 21 progressed precursor patients with a median time of progression of six years. From the 21 patients, four patients had no variation in one of the 81 studied genes. Interestingly, in 16 of the 17 other patients, at least one variant present in MM was also detected in its precursor BM, even years before progression. Here, the variants were present in the pre-stage at a median of 62 months before progression to MM. Studying these paired BM samples contributes to the knowledge of the evolutionary genetic landscape and provides additional insight into the mutational behavior of mutant clones over time throughout progression.

## 1. Introduction

Multiple myeloma (MM), also known as plasma cell (PC) myeloma or Kahler’s disease, is characterized by the clonal expansion of PCs in the bone marrow (BM). This second most common hematological malignancy is consistently preceded by one or more premalignant precursor conditions [1]. Monoclonal gammopathy of undetermined significance (MGUS) is an asymptomatic pre-stage, with approximately 1% of the patients annually progressing to MM. Smoldering multiple myeloma (SMM) is a premalignant condition defined by a higher disease burden, with the annual risk for progression of 10% in the first five years, 3% per year for the next five years and finally 1% per year for the subsequent years [2]. Both myeloma precursor conditions are caused by the monoclonal proliferation of PCs in the BM resulting in a detectable serum monoclonal (M)-protein in the blood and/or increased percentage of PCs in the BM. These myeloma precursor conditions are present in 3.5% of the population over 50 years [3,4]. Importantly, the pre-stages are completely asymptomatic without any evidence of the characteristic MM-specific end-organ damage or CRAB (hypercalcemia, renal failure, anemia, bone lesions) features.

Current parameters and models for risk stratification in myeloma precursor disease are based on rather indirect measures of the disease burden (e.g., M-protein levels, percentage of BM PCs and/or free light chain (FLC) ratios), which leads to limitations in risk assessment [5,6]. Moreover, reliable biomarkers to individually predict which patients will progress to MM and which will not are lacking [7]. The current standard of care and clinical management of patients with myeloma precursor conditions is based on the “watch and wait” strategy, involving observation and clinical follow-up without treatment until progression to the overt malignancy MM [8,9,10,11,12,13]. Even though current consensus guidelines are recommending indefinite follow-up of MGUS and SMM patients, data regarding optimal clinical management of these patients are not available [4,14,15,16]. Moreover, the majority of MM malignancies are still diagnosed only after signs of end-organ damage or presence of MM-related complications (e.g., CRAB) [13].

When evaluating currently used measures (e.g., M-protein levels or FLC ratios), only half of the precursor patients showed progressively increasing parameters prior to MM diagnosis, while the other patients had largely stable levels until the onset of the symptomatic MM disease. Moreover, a low disease burden based on current parameters does not specifically indicate a longer time to progression or to no progression at all [17,18]. These findings emphasize the importance of identifying and including other markers that would better reflect the correct disease biology and predict progression.

Besides clinical parameters, myeloma-related FISH is used in current routine diagnostic testing as an initial evaluation to investigate the specific cytogenetics and copy-number variants (CNVs) [19]. However, these techniques do not include the detection of other genetic lesions such as single nucleotide variants (SNVs) [20,21]. As demonstrated by next generation sequencing (NGS), somatic point mutations not only form the genetic landscape of MM but also of the pre-stages MGUS and SMM. SNVs in genes of the MAPK, DNA repair, NF-KB and cell cycle pathways are frequently involved and are considered to have relevance for disease progression. Some genes were found to have clonal alterations, while others were more frequently subclonal [5,22,23,24,25]. However, the SNVs and dysregulated pathways were mostly described in individual patients in separate disease stages. Even though the evolution of genetics during the progression from precursors to MM is described by comparing the different disease entities, it is questionable whether these results reflect the real situation within one patient. In fact, only a limited number of completed studies with mostly a limited number of included patients used paired follow-up samples from the same patient and report the behavior of variants over time during disease progression [23]. As also addressed by Dutta et al., these initial small-cohort bulk sequencing studies are very informative and important but still insufficient to generalize across all MM cohorts. More serial samples from larger cohorts of patients with longer follow-up durations are therefore needed [26]. Additionally, not all dysregulated pathways nor their clinical consequences and relevance have been completely understood [27].

The continuum between MGUS, SMM and MM provides the opportunity to investigate both the genetic hierarchy and clonal evolution in the progression. However, several obstacles impede the collection of serial follow-up samples during progression of the same patient: (i) the incidental diagnoses of asymptomatic myeloma precursor conditions, (ii) the invasiveness of BM sampling, (iii) the low percentages and numbers of aberrant PCs in the BM samples resulting in limited DNA availability and (iv) the rather non-uniform and irregular precursor disease follow-up. These limitations are especially valid for obtaining serial samples from low and intermediate risk myeloma precursor disease towards the progression to MM [28,29,30]. Therefore, it remains very difficult to elucidate and define the role of genetic events in relation to the malignant transformation and define its potential role as a biomarker in the prediction of the risk of progression to MM.

To our knowledge, few papers have described the genetic analyses and underlying molecular mutational mechanisms of paired samples from the same individuals progressing from myeloma precursor conditions to MM with NGS. However, while this unique “within patient” approach can lead to additional insights into the disease pathogenesis, current studies of serial samples mostly described the evolution from (high-risk) SMM patients to MM with a relatively short median time to progression [23,24,29,31,32,33]. Since it is currently impossible to predict if and when a pre-stage will progress to MM, a more biologically oriented strategy with molecular profiling of the BM samples could possibly help to answer this question. The inclusion of genetic lesions as parameters during stratification may potentially improve the risk prediction models for progression [21].

In this study, we developed a capture-based targeted sequencing panel in order to study the behavior of SNVs and short insertions and deletions (indels) in myeloma-related genes in paired samples from 21 low and intermediate risk precursor patients in their progression to MM. The Clinical Biobank Jessa Hospital and University Biobank Limburg (UBiLim) [34,35,36] contained a large hematological collection of paired BM smears at different time points in the progression to MM, circumventing aforementioned obstacles in collecting serial follow-up samples and allowing us to study the genetic evolution over time within one patient.

## 2. Materials and Methods

### 2.1. Samples

This study involved a retrospective collection of biobank-stored samples. The May–Grünwald–Giemsa (MGG)-stained BM smears used in this study were provided by the Clinical Biobank of the Jessa Hospital and University Biobank Limburg (UBiLim) [34,35,36]. Samples and data were obtained and managed in good clinical practice, in accordance with the Declaration of Helsinki. The study was approved by the medical ethics committees of the Jessa Hospital and Hasselt University (Belgium). Overall, 21 patients with at least two serial BM samples, one in pre-stage and one in newly diagnosed, untreated MM phase, were involved in this study. For each stage, the patient was diagnosed with MGUS, SMM or MM, according to current guidelines. The precursor condition MGUS was characterized by <3 g/dL M-protein, <10% clonal PCs on BM biopsy and absence of CRAB criteria and myeloma-defining events [37,38]. For SMM, diagnosis was based on following criteria: <3 g/dL M-protein, <10% clonal PCs on BM biopsy and absence of CRAB criteria and myeloma-defining events [37,39]. No precursor condition was ever treated.

### 2.2. DNA Extraction

Genomic DNA (gDNA) was manually extracted from the archival May–Grünwald–Giemsa (MGG)-stained BM smears using the QIAamp DNA Micro Kit (Qiagen, Hilden, Germany), as previously described [40]. The molecular usability of DNA extracted from these stained BM smears has been proven to be qualitatively and quantitatively fit-for-purpose for reliable downstream analyses including NGS [40].

### 2.3. Targeted Panel Design

The custom targeted sequencing gene panel was designed using the DesignStudio software tool (Illumina, San Diego, CA, USA) and included coding exons or hotspot positions of 81 genes (*ACTG1*, *ARID1A*, *ARID2*, *ATM*, *BCL7A*, *BHLHE41*, *BIRC2*, *BIRC3*, *BRAF*, *BTG1*, *CCND1*, *CDKN1B*, *CDKN2A*, *CDKN2C*, *CRBN*, *CUL4A*, *CUL4B*, *CYLD*, *DIS3*, *DNMT3A*, *DTX1*, *DUSP2*, *EGR1*, *FAM46C*, *FGFR3*, *FUBP1*, *HIST1H1B*, *HIST1H1D*, *HIST1H1E*, *HIST1H2BK*, *IDH1*, *IDH2*, *IGLL5*, *IKBKB*, *IKZF1*, *IKZF3*, *IRF1*, *IRF4*, *KDM6A*, *KLHL6*, *KMT2D*, *KRAS*, *LCE1D*, *LTB*, *MAF*, *MAFB*, *MAP3K1*, *MAX*, *MYC*, *MYD88*, *NFKB2*, *NFKBIA*, *NRAS*, *PABPC1*, *PIM1*, *POT1*, *PRDM1*, *PRKD2*, *PSMB5*, *PTPN11*, *RASA2*, *RB1*, *RFTN1*, *RIPK1*, *RPL10*, *RPL5*, *RPRD1B*, *RPS3A*, *SAMHD1*, *SETD2*, *SP140*, *STAT3*, *TBC1D29*, *TCL1A*, *TGDS*, *TP53*, *TRAF2*, *TRAF3*, *XBP1*, *ZNF292*, *ZNF462*). These target genes were selected for their known role in the separate disease entities of MM and its precursor conditions.

The design was based on the DNA target-enrichment approach reported by Bolli and colleagues [21] and supplemented with several other myeloma-related genes in relevant pathways from previous sequencing studies [41,42,43,44,45,46,47,48]. The total targeted region of our custom panel covered approximately 182 kb, and the mean amplicon size was 178 bp.

This custom myeloma panel was a capture-based approach. The library preparation was performed with the Illumina DNA Prep with Enrichment kit (Illumina), according to manufacturer instructions (FFPE Sample Recommendations). For input, double-stranded DNA of median 250 ng (ranging between 75 and 411 ng) was used. The enriched libraries were quantified using Qubit 3.0 (Life Technologies, Carlsbad, CA, USA) and qualified on a Bio-Analyzer (Agilent Technologies, Santa Clara, CA, USA) in order to assess successful enrichment and amplification. Four (precursor samples) or five (MM samples) pre-enriched libraries were pooled by mass for capture-bead-based hybridization. The pooled and captured libraries were then pooled per two for paired-end sequencing on a MiSeq flowcell with V2 chemistry at a final loading concentration of 7 to 8.7 pM. The PhiX control library (Illumina) was spiked in each run at a final concentration of 12.5 pM to estimate the sequencing error rate, as described in the manufacturer’s protocol.

After data analysis as described below, higher coverage sequencing was required for a number of samples. When a variant detected in the MM phase was not (significantly) found in the precursor BM of that patient, these precursor BM samples were selected for deeper sequencing. The pre-enriched libraries were then pooled per two for hybridization and sequenced on a MiSeq V2 flowcell with a final loading concentration of 8 to 8.7 pM.

### 2.4. Data Analysis and Variant Classification

Data were automatically analyzed in the Local Run Manager tool (Illumina) on the MiSeq instrument (Illumina) using the DNA Enrichment method (Illumina) and standard settings including a variant allele frequency (VAF) ≥ 1%. For each individual sample, the nucleotide sequences with their base quality scores in text format file (Fastq), the binary alignment map file (Bam) and variant calling file (Vcf) were generated.

Alignment was performed against the human genome reference sequence GRCh37/Hg19. For visualization of the read-alignment status, the Bam files were loaded in Integrative Genomics Viewer (IGV_2.9.4; The Broad Institute, Cambridge, MA, USA). For variant annotation and filtering, the Vcf files were imported into VariantStudio (Illumina). In VariantStudio, the exonic nonsynonymous as well as loss-of-function (stop-gain, frameshift, splice site) variants with coverage higher than 30 were filtered [49,50]. All filtered variants with a VAF of 1% or higher were assessed. If a particular SNV was detected in the MM phase but not in the pre-stages of that patient, these events were manually inspected in IGV. By checking if and in which frequencies the variants were present, it was verified that these variants were not due to duplicates, artefacts or background noise. To exclude sensitivity issues, the significant occurrence of a variant was verified. The frequency of the presence of that particular variant was compared to its frequency in 10 unrelated sequenced BM smears from the other patients. Only if the frequency of the variant was higher than three standard deviations of the mean of those 10 random samples was the variant defined as significantly detected.

For some patients, a variant present in the MM phase was not (significantly) detected in its precursor BM. In order to exclude sensitivity issues and to reflect the real biological situation, these precursor samples were selected for deeper sequencing. These NGS data were analyzed in the same way as mentioned previously. Deeper sequencing increases the limit of detection and allows the detection of any potentially missed variants.

Generally, the sequencing depth of our NGS assay was intended to be sufficiently high to detect low burden variants. The classification and annotation of the somatic variants were performed according to the Belgian guidelines [51]. All variants classified as pathogenic, likely pathogenic or variant of unknown significance were included for further analysis.

## 3. Results

### 3.1. Patient Characteristics

Targeted sequencing was performed on MGG-stained BM smears of 21 patients progressing from myeloma precursor conditions to MM (Table S1). Of these patients, 48% were women and 52% men. All patients had at least two BM smears: one in the precursor state and one in the newly diagnosed, untreated MM phase. For several patients, multiple samples from their precursor phase were available. In total, 68 samples were sequenced, with a mean of three samples per patient (ranging from two to seven BM smears per patient).

Most patients (18/21) had IgG as the M-protein, two IgAs and only one light chain. The median age at diagnosis of the myeloma precursor conditions and MM was 67 and 73 years, respectively. All patients progressed to MM with a median time to progression of 72 months, ranging from 6 to 166 months (almost 14 years). Of the 21 patients studied, 19 patients were in the MGUS phase at the first available sample, while only two were initially diagnosed as SMM. Out of 10 patients with more than two follow-up samples and starting with MGUS, six patients first passed through SMM prior to MM diagnosis. Based on currently used parameters, all myeloma precursor conditions were classified as low or intermediate risk [52,53].

### 3.2. Run Characteristics

Generally, the mean coverage of the targeted regions was 636 reads. On average, 99%, 96% and 86% of all targeted positions had a coverage of more than 30, 150 and 300 reads, respectively. However, for the deeper sequencing runs, the mean coverage of the targeted regions was 3410×. The percentage of base calls with a quality score of at least Q30 of each of the MiSeq runs was always higher than 90%, indicating high quality runs.

### 3.3. Somatic Mutations

The paired samples from patients progressing from a PC pre-stage to MM provide a unique insight into the role of SNVs and short indels and their behavior over time. Instead of examining the separate disease entities, the mutational profile within the patient was investigated. Moreover, the myeloma precursor conditions were defined as low or intermediate risk by current stratification [52,53], revealing the genetic architecture of somatic variant in the pre-stage with low proliferative PCs in the BM. In the 21 samples with BM PC percentages ranging from 6% to 65% in the MM phase, a total of 29 variants (SNVs or indels) were detected in 20 different genes (Table S2). Generally, a mean of 1.4 variants per patient was detected, classified as either variant of unknown significance, likely pathogenetic or pathogenic [51]. In four patients, no genetic aberration was detected in one of the assessed 81 genes, and these are henceforth not further discussed. The other 17 patients had at least one gene variant detected in the MM phase. No somatic mutations were identified in the pre-stage which were not present in the MM phase. After the initial sequencing of all samples, three patients had SNVs in their MM BM smears that were not found in the BM smears sampled in their precursor phase(s).

For the other 14 patients, the MM variant was detected in at least one precursor BM sample. Some patients had the variant detected only in a later precursor stage but not in any earlier available precursor samples. To exclude the possibility that the reason for the absence of variants was NGS sensitivity issues, those specific precursor samples were sequenced at about four- to five-times higher coverages. After deeper NGS analyses, there was only one patient in which no MM variants were detected in the precursor phase while present in their MM BM. The other 16 patients had at least one variant present in MM that was also detected in its precursor BM. Moreover, several variants previously not (significantly) detected in the first and earlier precursor BM samples were found after deeper sequencing.

Variants of unknown significance (VUS) were found in *BCL7A*, *DIS3*, *FAM46C*, *HIST1H1E*, *IDH1*, *IKBKB*, *IRF4*, *MAX*, *RASA2*, *SP140* and *XBP1*. Variants in *ARID2*, *BRAF*, *DNMT3A*, *FAM46C*, *KRAS*, *PTPN11*, *SETD2*, *TP53* and *HISTH1D* were classified as likely pathogenic, while KRAS and NRAS had SNVs with a pathogenic classification.

Additionally, clonal hematopoiesis-associated variants were detected in *IDH1* and *DNMT3A* genes. These mutations were based on higher than expected VAFs in precursor stages, similar to MM. These variants can be linked to clonal hematopoiesis of indeterminate potential (CHIP).

**Figure 1:**
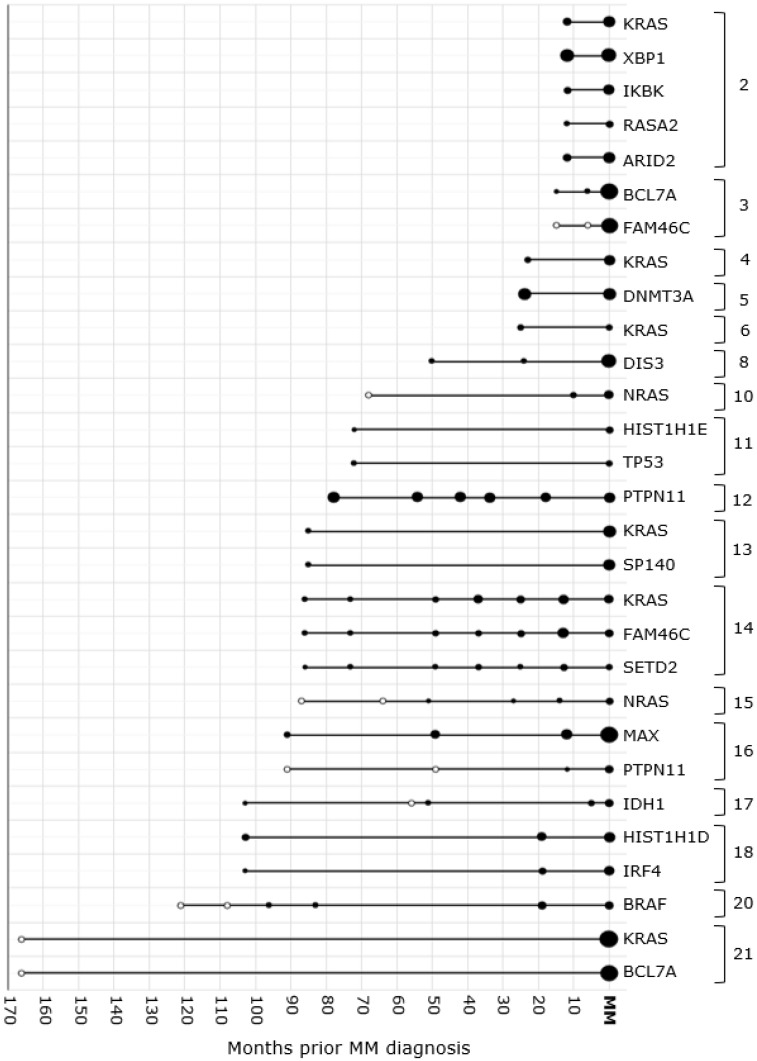
Overview of detected variants in the progression from precursor stage(s) to MM. In 17 patients, the MM BM smear had at least one variant. In 16 of the 21 patients, variants detected in the MM phase were already present in the precursor stage. A significantly detected variant is indicated by a black dot. The size of the dot is representative for the VAF. In BM samples in which the variant was not detected, the dot is open. The time on the x-axis is given in months prior to the MM diagnosis. Mutations found in the same patient were indicated with an accolade and patient number. BM: bone marrow; VAF: variant allele frequency; MM: multiple myeloma.

In 16 of the 17 patients with at least one variant in their MM sample, the variant was already detected in its asymptomatic pre-stage. In total, 26 variants detected in MM were also detected and present in at least one pre-stage sample of the corresponding patient (Table 1). The detected SNVs were present in the precursor condition at a median time of 62 months (range 10–103) prior the progression into MM (Figure 1).

## 4. Discussion

In this study, we were able to analyze archival diagnostic MGG-stained BM smears from progressing low or intermediate risk myeloma precursor conditions. These serial unsorted BM samples in the progression to MM were studied with a custom targeted sequencing panel, including 81 myeloma-associated genes. In this unique approach, we were able to show that most of the variants detected in MM were already present in the precursor phases, even many years before progression.

A healthy PC transforms into a malignant myeloma cell through a heterogeneous multistep process. The genetic make-up characteristics of MM, being hyperdiploidy (trisomies of odd-numbered chromosomes) or non-hyperdiploidy (IGH translocations), are already present at the early myeloma precursor conditions although at lower frequencies. These alterations are thought to be the primary events required but not sufficient to drive MM development since many MGUS and SMM patients carry these alterations for decades without any signs of progression. Therefore, secondary alterations are believed to be required to trigger the progression to MM. However, the specific and direct mechanisms driving the transformation to MM are still unknown [5,54,55,56,57].

Next to the hyperdiploidy status, several other genetic factors are known to contribute to shaping the disease pathology of MM and its precursors. With the advent of NGS, the knowledge on the genomic landscape has been significantly increased and provided additional important insight into the disease. Nevertheless, mutation screening has not yet been implemented in standard clinical workflows [27].

Currently used clinical parameters for risk assessment are useful tools but not suitable to determine the individual progression risks of myeloma precursors. Moreover, a consensus on which parameters to use and on the most optimal clinical management strategy is also missing [4,7,14,15,16]. Despite the serial follow-up and laboratory testing of these precursor patients, most of the MM cases are still diagnosed after clinical presence of MM-related symptoms [13]. Hence, clinicians must stay attentive for the presence of myeloma-related end-organ damage and CRAB features [18,58]. Therefore, a more biology-oriented strategy with molecular markers, e.g., the incorporation of a spectrum of genetic lesions, may lead to significant improvements in individual risk assessments for progression.

The mutational profile and behavior of SNVs and short indels in the progression to MM could reveal potential driving events. Current studies with paired samples mostly used expression and cytogenetic analysis to study the progression [59,60,61,62,63,64]. So far few NGS studies have used serial follow-up samples from the same individuals progressing from myeloma precursor conditions to MM [23,24,29,31,32,33]. However, these studies analyzed almost exclusively follow-up samples from (high-risk) SMM patients to MM with a relatively short median time to progression. Moreover, they only sequenced a rather small number of progressing patients [5,24,29,31,32], nevertheless revealing relevant insights into the genetic patterns and mechanisms of progression. Interestingly, the genetic profiles of the progressed precursors were found to be similar to what has been described for MM. However, this similarity could potentially be due to the high-risk characteristics of the studied precursors and/or the relatively short time to progression [5,23,24,29,31,32,33].

In this study, a unique retrospective collection of paired BM samples was used, which tackled the aforementioned sampling problems and made it possible to study the mutational profile of SNVs and indels within a patient over time during its evolution from pre-stage to MM. Somatic mutations affecting MAPK/ERK (e.g., *KRAS*, *BRAF*, *PTPN11*, *RASA2*), NF-KB (e.g., *NFKB2*), cell cycle (e.g., *CCND1*), DNA repair (e.g., *TP53*) and RNA metabolism (e.g., *DIS3*, *FAM46C*) signaling pathways were detected. Dysregulation of these pathways is an established mechanism in malignant PCs [5,41,54,55,56,65,66,67,68,69]. These SNVs have previously been shown to contribute to part of the genetic landscape of the myeloma precursor conditions MGUS and SMM as well [5,8,22,23,24,25,70]. Mutations in epigenetic enzyme-related genes (e.g., *ARID1A*, *HIST1HE*, *SETD2*) also occurred in these PC disorders. The mutational dysfunction of epigenetic regulators related to histone acetylation, DNA methylation and chromatin remodeling contribute to shaping the complex molecular make-up of these myeloma patients [71,72,73,74].

The clonal hematopoiesis-associated mutations were based on higher than expected VAFs in precursor stages. The association of CHIP variants in PC disorders, in particular MM, has also been previously described [75,76].

Since aberrant AID activity is one of the known principal mutational processes in MM and its early stages [24,31,55,77,78,79], we investigated the potential enrichment for early mutations in AID targets in these paired samples based on the reported catalog of canonical AID target genes [80]. Some AID targets were part of our custom panel. However, since this catalog is based on previous observations in non-Hodgkin lymphomas, it is possible that not all of these genes are targeted by AID in MM too, or other AID targets in MM might not be included. However, AID seems to be an early common driver mutational process, already present in the myeloma precursor conditions. This process contributes to the generation of the mutational spectrum of the transformed post-germinal center B-cell, which gives rise to the myeloma clone [31,78]. Known AID targets (e.g., *IRF4*, *XBP1*) were found to be mutated in some cases of our study cohort of progressed precursors as well, confirming the assumption that aberrant AID activity contributes to shaping the genomic landscape and the initiation of MM [31,78,80].

A mutation was also found in *MAX*, the binding factor of the oncogenic transcription factor MYC. Recently, *MAX* was also observed to be a target of mutation in malignancies such as MM. However, the oncogenic consequence and functional significance of these SNVs are still not well understood [81,82]. Lastly, mutations were detected in genes whose function was still largely unknown such as *FAM46C* and *BCL7A*. Although frequently mutated in MM, the effect on and association with the disease is unclear [83,84,85,86].

The variants identified in this study were heterogeneous and could not be reduced to one or a few specific pathways, underlining the complex and heterogeneous characteristics of MM. In fact, no gene predicted the progression since the majority of variants in MM phase samples could already detected (at lower VAFs) in the BM smears sampled in the precursor phase, even many years before progression. Nevertheless, a similar study on serial samples from stable non-progressing myeloma precursor patients would be a potential added value in order to validate this hypothesis and to determine their direct prognostic value in predicting the progression or the risk. Unfortunately, the availability of these serial samples and associated ethical issues will remain the main obstacle to initiating such a study.

Our data thus may indicate that the early presence of variants represents a prognostic indication for progression. However, the finding that even in the presence of mutations such as *TP53* and *KRAS* the myeloma precursor conditions may still take years to progress is remarkable. These findings could possibly lead to questions regarding the role of mutations in dictating progression, since time to progression is apparently not impacted by any single or subtle nucleotide changes. These data may suggest a limited value of SNVs and/or indels for the prediction of progression in the short term, which can impact experimental approaches in prospective mutation detection (i.e., in cell-free DNA). Moreover, other important factors playing a role in the disease progression should potentially be taken into account, i.e., the early and specific immune recognition by interactions between PCs and the BM microenvironment [87].

Generally, current knowledge is still insufficient to generalize across all precursor and MM cohorts [26]. More serial samples from larger cohorts of patients with longer follow-up periods are needed. Before incorporating such approaches in the follow up of MGUS/SMM patients to efficiently sketch and detect (high risk) progressors, more studies need to confirm and/or extend our data. The development of rigorous and accurate disease-risk tools requires additional high-quality health data, adequate technically robust performance metrics and accurate, reliable, reproducible and generalizable data on the risk assessment of disease progression. Moreover, specific criteria will be required to assess the effectiveness of such approaches to predict MGUS/SMM progression and risk stratification of these precursor patients. Lastly, feasibility studies and implementation of proof of concept, i.e., in clinical trials, is required for demonstrating the improved performance of such a tool in comparison to the standard of care.

Our study also has some limitations, however. First, our analysis was challenged by the mandatory use of unsorted BM smears, which inherently only harbor a very low percentage of aberrant PCs, likely with (sub)clonal heterogeneity. In such a set-up, a high coverage and high sensitivity is required to detect (minimal) relevant mutations [43]. Since somatic mutations are present at different frequencies, it was important to define the lowest VAFs at which variants could be confidently and reliably detected. By sequencing at high depth, we demonstrated the usefulness of a targeted sequencing approach to detect potentially relevant mutations in unsorted BM samples from myeloma precursor conditions.

Second, the recommended use of a targeted panel to yield the required high coverage simultaneously led to the downside that this panel only interrogates a specific selection of regions that is based on the current knowledge and thereby might not reflect the full biological basis of the disease [88,89]. It harbors the coding or hotspot regions of 81 myeloma-related genes only and consequently cannot detect mutational signatures, CNVs and structural variations, or even more complex events such as chromothripsis, which have been reported in MM and progressed precursors recently [90]. Targeted NGS restricts the potential to investigate other genomic events. Moreover, it is possible that not all causative molecular variation in MM has been fully identified, and its clinical significance is also not completely understood. Other variants, not included in the panel, are potential additional factors which are pathologically pushing the aberrant PC clone to a malignant fate. Nevertheless, compared to more comprehensive NGS approaches, targeted sequencing is still the most favorable method for NGS-based screenings, given the low amounts of DNA required, the higher depth of coverage, the easier/less demanding bioinformatic analysis, the shorter turnaround time and the much lower cost [50].

The use of sorted aberrant PCs is recommended in the future. Given the low percentage of BM PCs, it will suffer from low amounts of DNA. Notably, we have successfully used a low-input method for whole genome sequencing (WGS) [90], but this method should first be optimized for the use of targeted sequencing.

## 5. Conclusions

In summary, the mutational profile underlying the progression from low or intermediate risk myeloma precursor conditions to MM was investigated. Most of the variants detected in the MM phase were already present in the pre-stage. With the use of targeted sequencing, we were also able to detect variants at lower frequencies. Progressed precursors seem to share the same mutational profile, even years before actual progression. Sequencing of these paired samples contributed to additional insights into the mutational behavior of mutant clones over time throughout the progression to MM.

## Figures and Tables

**Table 1:** Patients, their characteristics and detected variants. All 21 patients with at least two serial BM smears were sequenced with the targeted panel including 81 myeloma-related genes. In this table, the precursor stage, gender, age of diagnosis (years), time to progression (months), serial samples and detected variants are shown for all patients.

Patient	Precursor Stage	Gender	Age Diagnosis (Year)		Time to Progression (Months)	Number of Serial Samples	Mutations ^a^ Detected		Variants Detected in MM
			MGUS/SMM	MM			Precursor	MM	
1	MGUS	F	72	73	6	2	nd	nd	No variants
2	SMM	F	85	86	12	2	X	X	*ARID2*, *RASA2*, *IKBK*, *XBP1*, *KRAS*
3	MGUS	F	80	81	15	3	X	X	*FAM46C*, *BCL7A*
4	MGUS	M	56	58	23	2	X	X	*KRAS*
5	MGUS	M	78	80	24	2	X	X	*DNMT3A*
6	SMM	M	74	76	25	2	X	X	*KRAS*
7	MGUS	F	67	70	39	2	nd	nd	No variants
8	MGUS	F	80	84	50	3	X	X	*DIS3*
9	MGUS	M	68	73	54	2	nd	nd	No variants
10	MGUS	M	65	71	68	3	X ^b^	X	*NRAS*
11	MGUS	F	56	62	72	2	X	X	*TP53*, *HIST1HE*
12	MGUS *	M	67	73	78	6	X	X	*PTPN11*
13	MGUS	M	64	71	85	2	X	X	*SP140*, *KRAS*
14	MGUS *	F	70	77	86	7	X	X	*SETD2*, *FAM46C*, *KRAS*
15	MGUS *	F	54	61	87	6	X ^b^	X	*NRAS*
16	MGUS *	M	62	70	91	4	X	X	*PTPN11*, *MAX*
17	MGUS	F	54	63	103	5	X	X	*IDH1*
18	MGUS *	F	69	77	103	3	X	X	*IRF4*, *HISTH1D*
19	MGUS	M	50	59	105	2	nd	nd	No variants
20	MGUS *	M	67	77	121	6	X ^b^	X	*BRAF*
21	MGUS	M	62	76	166	2	nd	X	*BCL7A*, *KRAS*

* Patients with MGUS first passing through SMM prior to MM diagnosis. ^a^ A somatic mutation detected in at least one of the 81 tested MM-related genes. ^b^ Mutation not detected in the first precursor sample(s). M: male; F: female; nd: none detected.

## Data Availability

The data presented in this study are available on request from the corresponding author.

## Supplementary Materials

The following supporting information can be downloaded at: https://www.mdpi.com/article/10.3390/cancers14041035/s1, Table S1: Percentage of PCs in the precursor BM smears; Table S2: Detailed overview of the 29 identified variants.
