# Supplementary material for: The Dynamics of Nucleotide Variants in the Progression from Low–Intermediate Myeloma Precursor Conditions to Multiple Myeloma: Studying Serial Samples with a Targeted Sequencing Approach
Source: Cancers (Basel). 2022 Feb 18;14(4):1035. doi: 10.3390/cancers14041035 (PMC8870380; doi:10.3390/cancers14041035)
Supplement: Supplementary file 1 [file cancers-14-01035-s001.zip › cancers-1541138-supplementary.pdf]

cancers

MDPI

# Supplementary Materials: The Dynamics of Nucleotide Variants in the Progression from Low–Intermediate Myeloma Precursor Conditions to Multiple Myeloma: Studying Serial Samples with a Targeted Sequencing Approach

Bénedith Oben, Charlotte Cosemans, Ellen Geerdens, Loes Linsen, Kimberly Vanhees, Brigitte Maes, Koen Theunissen, Bert Cruys, Marta Lionetti, Ingrid Arijs, Niccolò Bolli, Guy Froyen and Jean-Luc Rummens

**Table S1.** Percentage of PCs in the precursor BM smears. For each patient, the start precursor phase, the total number of serial precursor samples and the percentage of PCs in the BM smears chronologically ordered were indicated.

| Patient | Precursor Stage | Number of Serial Precursor Samples | % PCs in BM Smear, Chronologically over Time |
|---|---|---|---|
| | | | Precursor Phase |
| 1 | MGUS | 1 | 6 |
| 2 | SMM | 1 | 12 |
| 3 | MGUS | 2 | 3–1 |
| 4 | MGUS | 1 | 5 |
| 5 | MGUS | 1 | 5 |
| 6 | SMM | 1 | 12 |
| 7 | MGUS | 1 | 3 |
| 8 | MGUS | 2 | 4–2 |
| 9 | MGUS | 1 | 4 |
| 10 | MGUS | 2 | 6–9 |
| 11 | MGUS | 1 | 3 |
| 12 | MGUS* | 5 | 6–9–17–8–12 |
| 13 | MGUS | 1 | 5 |
| 14 | MGUS* | 6 | 6–6–10–12–17–16 |
| 15 | MGUS* | 5 | 12–12–8–13–19 |
| 16 | MGUS* | 3 | 7–12–30 |
| 17 | MGUS | 4 | 5–8–7–12 |
| 18 | MGUS* | 2 | 9–19 |
| 19 | MGUS | 1 | 5 |
| 20 | MGUS* | 5 | 6–3–3–4–10 |
| 21 | MGUS | 1 | 1 |

US: Variant PC: plasma cells; BM: bone marrow.

**Table S2.** Detailed overview of the 29 identified variants. A total of 29 variants were detected in 20 different genes. Each identified somatic variant was annotated and classified as Pathogenic, Likely Pathogenic or Variant of Unknown Significance, according to the Belgian guidelines[1]. For each variant, the location (chromosome and coordinate) and HGVS coding and protein sequence name was showed.

| Gene | Classification | HGVSc | HGVSp | Chr | Coordinate |
|---|---|---|---|---|---|
| *ARID2* | LIKELYPATHOGENIC | NM_152641.2:c.3410C > A | NP_689854.2:p.Ser1137Ter | 12 | 46245316 |
| *BCL7A* | US | NM_020993.3:c.36C > A | NP_066273.1:p.Ser12Arg | 12 | 122460033 |
| *BCL7A* | US | NM_020993.3:c.74T > C | NP_066273.1:p.Ile25Thr | 12 | 122460071 |
| *BRAF* | LIKELYPATHOGENIC | NM_004333.4:c.1780G > A | NP_004324.2:p.Asp594Asn | 7 | 140453155 |
| *DIS3* | US | NM_014953.3:c.1663_1668delGTGGAC | NP_055768.3:p.Val555_Asp556del | 13 | 73345220 |
| *DNMT3A* | LIKELYPATHOGENIC | NM_175629.2:c.939G > A | NP_783328.1:p.Trp313Ter | 2 | 25470535 |
| *FAM46C* | US | NM_017709.3:c.275A > G | NP_060179.2:p.Asp92Gly | 1 | 118165765 |
| *FAM46C* | LIKELYPATHOGENIC | NM_017709.3:c.584_585delAT | NP_060179.2:p.Tyr195Ter | 1 | 118166072 |
| *HIST1H1D* | LIKELYPATHOGENIC | NM_005320.2:c.556G > A | NP_005311.1:p.Ala186Thr | 6 | 26234606 |
| *HIST1H1E* | US | NM_005321.2:c.139G > A | NP_005312.1:p.Ala47Thr | 6 | 26156757 |
| *IDH1* | US | NM_005896.2:c.940C > T | NP_005887.2:p.Arg314Cys | 2 | 209104638 |
| *IKBKB* | US | NM_001556.2:c.2033C > G | NP_001547.1:p.Ala678Gly | 8 | 42183534 |
| *IRF4* | US | NM_002460.3:c.316G > T | NP_002451.2:p.Asp106Tyr | 6 | 394920 |
| *KRAS* | PATHOGENIC | NM_033360.2:c.38G > A | NP_203524.1:p.Gly13Asp | 12 | 25398281 |
| *KRAS* | PATHOGENIC | NM_033360.2:c.35G > A | NP_203524.1:p.Gly12Asp | 12 | 25398284 |
| *KRAS* | PATHOGENIC | NM_033360.2:c.436G > A | NP_203524.1:p.Ala146Thr | 12 | 25378562 |
| *KRAS* | LIKELYPATHOGENIC | NM_033360.2:c.176C > A | NP_203524.1:p.Ala59Glu | 12 | 25380282 |
| *KRAS* | PATHOGENIC | NM_033360.2:c.38G > A | NP_203524.1:p.Gly13Asp | 12 | 25398281 |
| *KRAS* | PATHOGENIC | NM_033360.2:c.35G > A | NP_203524.1:p.Gly12Asp | 12 | 25398284 |
| *MAX* | US | NM_002382.4:c.312G > T | NP_002373.3:p.Lys104Asn | 14 | 65543365 |
| *NRAS* | PATHOGENIC | NM_002524.4:c.35G > C | NP_002515.1:p.Gly12Ala | 1 | 115258747 |
| *NRAS* | PATHOGENIC | NM_002524.4:c.181C > A | NP_002515.1:p.Gln61Lys | 1 | 115256530 |
| *PTPN11* | LIKELYPATHOGENIC | NM_002834.3:c.1520C > A | NP_002825.3:p.Thr507Lys | 12 | 112926900 |
| *PTPN11* | LIKELYPATHOGENIC | NM_002834.3:c.181G > A | NP_002825.3:p.Asp61Asn | 12 | 112888165 |
| *RASA2* | US | NM_006506.2:c.622_626delAAGAA | NP_006497.2:p.Lys208AspfsTer13 | 3 | 141274689 |
| *SETD2* | LIKELYPATHOGENIC | NM_014159.6:c.4667_4670delTCAC | NP_054878.5:p.Leu1556GlnfsTer8 | 3 | 47155410 |
| *SP140* | US | NM_007237.4:c.265G > A | NP_009168.4:p.Val89Ile | 2 | 231102955 |
| *TP53* | LIKELYPATHOGENIC | NM_000546.5:c.574C > T | NP_000537.3:p.Gln192Ter | 17 | 7578275 |
| *XBP1* | US | NM_001079539.1:c.11T > C | NP_001073007.1:p.Val4Ala | 22 | 29196502 |

US: Variant of Unknown Significance; HGVSc: Human Genome Variation Society coding; HGVSp: Human Genome Variation Society protein; Chr: Chromosome.

## References

1. Froyen, G.; Le Mercier, M.; Lierman, E.; Vandepoele, K.; Nollet, F.; Boone, E.; Van der Meulen, J.; Jacobs, K.; Lambin, S.; Vander Borght, S.; et al. Standardization of Somatic Variant Classifications in Solid and Haematological Tumours by a Two-Level Approach of Biological and Clinical Classes: An Initiative of the Belgian ComPerMed Expert Panel. *Cancers* **2019**, *11*, 2030, doi:10.3390/cancers11122030.
